# Dietary Recombinant Phycoerythrin Modulates the Gut Microbiota of H22 Tumor-Bearing Mice

**DOI:** 10.3390/md17120665

**Published:** 2019-11-26

**Authors:** Hongtao Qi, Ying Liu, Xin Qi, Hui Liang, Huaxin Chen, Peng Jiang, Dongfeng Wang

**Affiliations:** 1 Laboratory of Food Chemistry and Nutrition, College of Food Science and Engineering, Ocean University of China, Qingdao 266003, China; qiqi6917@sina.com; 2 Food Science and Engineering Department, College of Life Sciences, Qingdao University, Qingdao 266071, China; 3 Laboratory of Cell and Molecular Biology, Basic Medical College, Qingdao University, Qingdao 266071, China; shenghua005@163.com; 4 Key Laboratory of Marine Drugs, Chinese Ministry of Education, School of Medicine and Pharmacy, Ocean University of China, Qingdao 266003, China; qxhin@163.com; 5 Department of Human Nutrition, College of Public Health, Qingdao University, Qingdao 266021, China; qdlianghui@126.com; 6 CAS Key Laboratory of Experimental Marine Biology, Institute of Oceanology, Chinese Academy of Sciences, Qingdao 266071, China; chenhx001@126.com; 7 Laboratory for Marine Biology and Biotechnology, Qingdao National Laboratory for Marine Science and Technology, Qingdao 266237, China; 8 Centre for Ocean Mega-Science, Chinese Academy of Sciences, Qingdao 266071, China

**Keywords:** recombinant phycoerythrin, anti-tumor and immunomodulatory effects, intestinal flora, high-throughput sequencing, microbial barrier

## Abstract

Normal intestinal flora is widely involved in many functions of the host: nutritional metabolism; maintenance of intestinal microecological balance; regulation of intestinal endocrine function and nerve signal transduction; promotion of intestinal immune system development and maturation; inhibition of pathogenic bacteria growth and colonization, reduction of its invasion to intestinal mucosa, and so on. In recent years, more and more studies have shown that intestinal flora is closely related to the occurrence, development, and treatment of various tumors. It is indicated that recombinant phycoerythrin (RPE) has significant anti-tumor and immunomodulatory effects. However, little is known about the mechanism of the effect of oral (or intragastric) administration of RPE on gut microbiota in tumor-bearing animals. In this study, using high-throughput 16S rDNA sequencing, we examined the response of gut microbiota in H22-bearing mice to dietary RPE supplementation. The results showed that the abundance of beneficial bacteria in the mice intestinal flora decreased and that of the detrimental flora increased after inoculation with tumor cells (H22); following treatment with dietary RPE, the abundance of beneficial bacteria in the intestinal flora significantly increased and that of detrimental bacteria decreased. In this study, for the first time, it was demonstrated that dietary RPE could modulate the gut microbiota of the H22 bearing mice by increasing the abundance of beneficial bacteria and decreasing that of detrimental bacteria among intestinal bacteria, providing evidence for the mechanism by which bioactive proteins affect intestinal nutrition and disease resistance in animals.

## 1. Introduction

Phycobiliproteins are light-harvesting chromoproteins found in red algae, blue-green algae, Cryptophyta, and a few dinoflagellates, and include phycoerythrin (PE), phycoerythrocyanin (PEC), phycocyanin (PC), and allophycocyanin (APC) [1]. Phycobiliproteins are not only a type of protein, but also excellent natural edible pigments [2]. They have many biological functions, such as anti-cancer [3], anti-inflammatory, and anti-oxidation [4,5]. Moreover, they can improve lymphocyte activity and enhance immunity [6]. Thus, phycobiliproteins can be used in the pharmaceutical industry or developed into healthy foods.

Currently, the direct extraction of phycobiliprotein from various algae is the main method for obtaining the protein. However, the extraction from natural algae is very complex and expensive [7], and these difficult-to-extract phycobiliproteins have lower activity. Thus, some researchers are assembling related genes in *Escherichia coli* to obtain phycobiliproteins with higher purity and better stability through biosynthesis [8]. The phycobiliprotein used in this study was recombinant phycoerythrin (RPE).

In recent years, with the development of genomics and molecular biology technologies, the relationship between intestinal microorganisms and the occurrence and development of diseases has attracted great attention [9,10,11,12]. The gastrointestinal tract is the largest microbial colonized organ in the body, as it maintains intestinal bacteria. The dynamic balance of the microflora can prevent the invasion of pathogens, thus ensuring the stability of the internal environment of the organism [13]. Normally, the composition and structure of intestinal flora are relatively stable, and the beneficial and detrimental bacteria in the intestine are in a relatively balanced state. Many factors affect the diversity of intestinal flora. Diet and antibiotic use will have certain effects on the diversity of intestinal flora, and especially diet is the main factor that affects their diversity [14,15]. The growth, reproduction, and change of intestinal microorganisms are closely related to the composition of bacterial flora in feces. Therefore, studying the diversity of bacterial flora in feces can help understand the changes in the intestinal flora.

In this study, H22 hepatocellular carcinoma cells were inoculated into the axilla of mice to construct a subcutaneous transplanted tumor model. The effects of RPE on the intestinal flora of tumor-bearing mice were investigated following intragastric administration and comparison with the normal control group, model group and cyclophosphamide (CP) control group.

## 2. Results and Analysis

### 2.1. Effect of RPE on Tumor Inhibition in Mice

**Figure 1:**
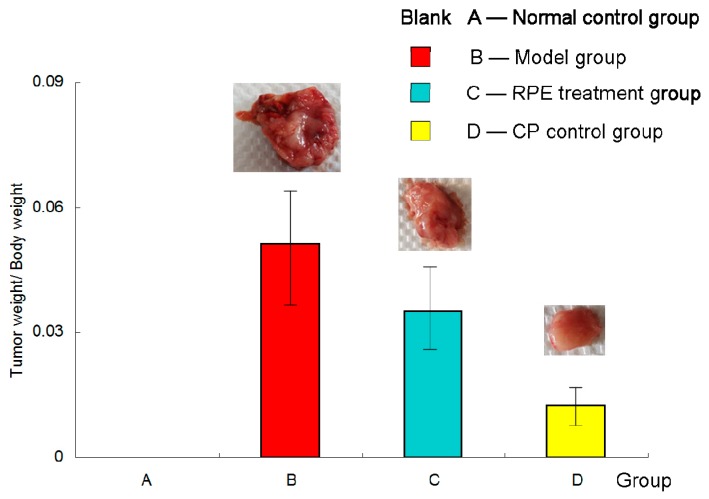
Tumor weight/body weight of the mice at the end of the experiment. Mice in group (**A**) (normal control group) were not inoculated with tumor cells. Mice in the (**B**), (**C**), and (**D**) groups were subcutaneously inoculated with 0.2 mL of an H22 cell suspension under the right forelimb axilla. The RPE treatment group (**C**) was administered RPE at 100 mg· kg bw^−1^·day^−1^ by gavage; the normal control group (**A**) and the model group (**B**) were i.g. administered with an equal volume of normal saline; the CP control group (**D**) was injected with cyclophosphamide (40 mg/kg) into the abdominal cavity every 2 days. Before the mice were killed, their body weight was measured for the last time. The tumors were dissected completely and weighed accurately. Effect of tumor inhibition was calculated by tumor weight/body weight.

Figure 1 showed the inhibition effect of recombinant phycoerythrin (RPE) on the H22 tumor-bearing mice. Group (A) was the normal control group and no tumor model was established in this group of mice. But for the sake of data integrity and convenience of comparison, group (A) was also presented in the figure, although being blank. Mice in the (B), (C), and (D) groups were subcutaneously inoculated with H22 tumor cells, and (B) and (C) were treated by intragastrical (i.g.) administration of physiological saline and recombinant phycoerythrin (RPE) solution, respectively; (D) was received intraperitoneal (i.p.) injection with cyclophosphamide (CP) as a positive drug control. As seen from the figure, the average ratio of (tumor weight/body weight) of the model group (B) was much higher than RPE treatment group (C) and the cyclophosphamide (CP) control group (D), and there was a significant difference (*p* < 0.05). In other words, both the RPE and CP treatment could obviously inhibit tumor growth. The tumor inhibition rates of RPE group and CP control group were 31% and 75%, respectively.

**Figure 2:**
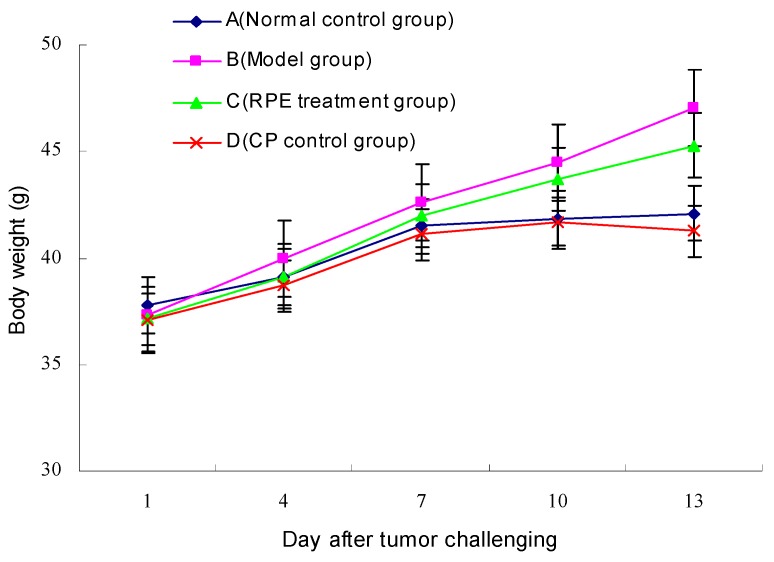
Body weight variations of the mice after tumor challenging. After a one-week adaptation period, all mice were randomly allocated into four test groups: normal control group (**A**), model group (**B**), RPE treatment group (**C**), and CP control group (**D**). Body weight was measured on the first day and then every 3 days after tumor challenging.

Body weight of the mice after tumor challenging was shown in Figure 2. In the first two days after inoculation, the average body weight of mice in each group was of no significant difference (*p* > 0.05); from the third day, the mice in the model group (B) began to develop tumors and their body weight increased faster. The tumors of the mice in the RPE treatment group (C) and the CP control group (D) appeared 2 days later than in the model group (B); thus, from the 5th day, the body weight of mice in the three tumor-bearing groups all increased rapidly. After the 7th day, with the treatment of RPE intragastric administration and CP intraperitoneal injection taking effect, the growth rate of body weight in RPE and CP groups was lower than that in the model group, especially in the CP group, which was even slightly lower than that in normal control group.

### 2.2. Effect of RPE on Intestinal Flora in Mice

#### 2.2.1. Sample Complexity

##### Rank-Abundance Curve of Microorganisms in Samples

**Figure 3:**
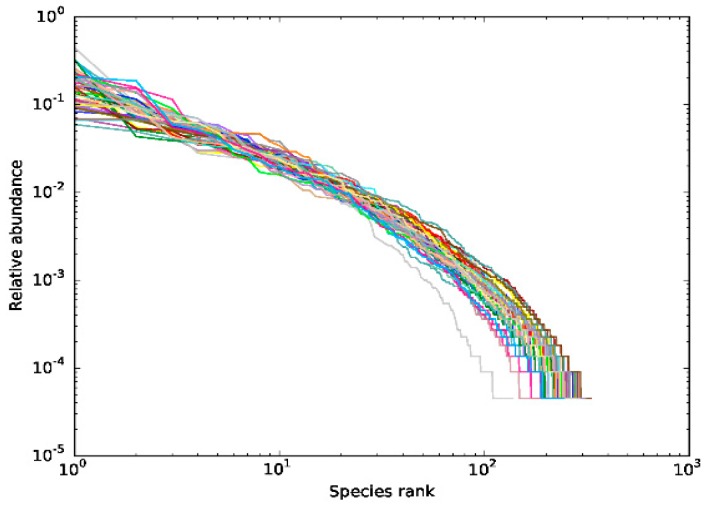
Rank abundance curve of bacterial OTUs derived from each sample. Different samples are represented by curves of different colors. The abscissa is the number rank sorted by OTU abundance, and the ordinate is the corresponding OTU abundance.

OTU (Operational Taxonomic Units) rank-abundance curve shows the diversity of samples, which can be explained by two aspects: species richness and evenness. The wider the span of the curve on the horizontal axis, the richer the species rank of the sample; the flatter the curve, the more uniform the species composition of the sample [5]. As shown in Figure 3, the horizontal direction of most of the curves is wide, showing that the species composition of the samples was rich; the vertical direction is flat and smooth (i.e., the slope of the curve is relatively gentle), indicating that the abundance distribution of the samples were even. Both the relatively high richness and evenness of the samples revealed that the diversity of the samples was rational and reasonable.

##### Dilution Curves of Microorganisms in Samples

**Figure 4:**
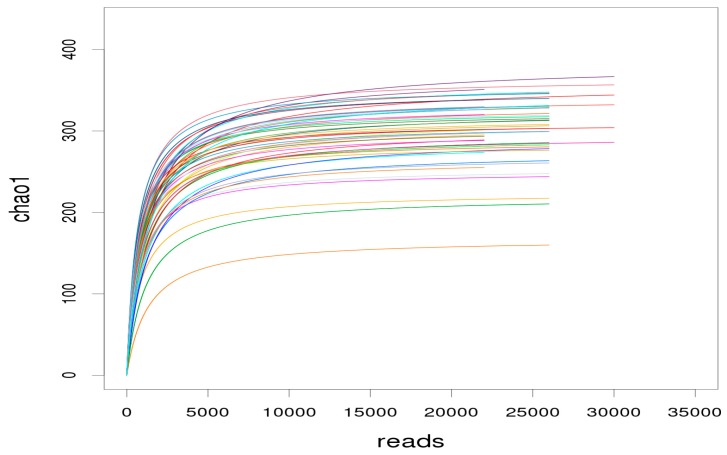
Dilution curve of the alpha diversity index for species abundance of samples. The horizontal axis represents the number of clean reads randomly extracted from a sample, and the vertical axis represents the alpha diversity index corresponding to the number of reads. Each curve in the figure represents a sample, presented in a different color.

The dilution curve is used to determine whether the sequencing depth is sufficient to cover all microbial species and indirectly reflect the species richness in the samples. When the curve flattens or reaches the plateau stage, it can be concluded that the sequencing depth has covered all species in the samples [16]. As shown in Figure 4, with the sequencing depth increasing, an inflection point appeared around a sequence number of 5000. As the sequencing depth increased further, the curves tended to be flat. When the number of sequences reached 10,000, the curves were in the plateau stage. In this study, the average sequencing depth of each sample was more than 10,000. It was concluded that the sequencing depth was sufficient to cover all bacterial species and enough to reflect the richness level, which also ensured that the data of sample sequencing in each group were reasonable.

#### 2.2.2. Diversity of Intestinal Flora

##### Alpha Diversity of Intestinal Flora

**Figure 5:**
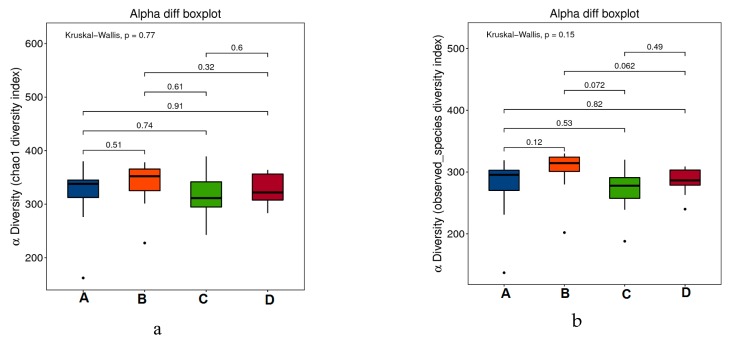

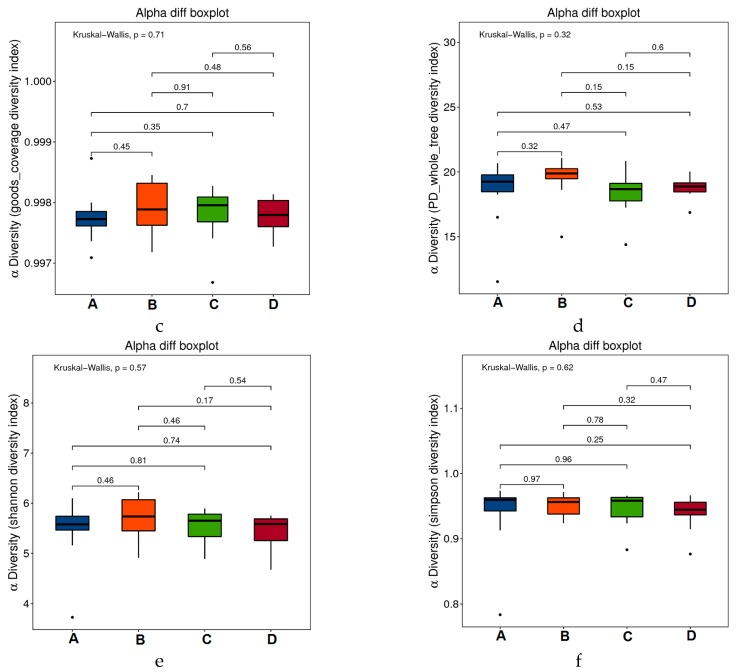
Alpha-diversity boxplots between the four groups, including Chao1 (**a**), Observed (**b**), Goods_coverage (**c**), PD_whole_tree (**d**), Shannon (**e**), and Simpson indexes (**f**).

Alpha diversity was analyzed to assess the species diversity of an individual sample and it included analysis of the Chao1 index, observed species index, PD_whole_tree index, goods-coverage index, Shannon index, and Simpson index. As shown in the Figure 5a–f, there was no significant difference in flora abundance and diversity among the groups (*P* values between the groups were marked, *p* > 0.05). Thus, during the experiment period, all the treatment: subcutaneous inoculation of H22 tumor cell (B), dietary RPE (C) and intraperitoneal injection of cyclophosphamide (D) did not change the diversity of the intestinal flora of mice in the groups significantly.

##### Beta Diversity of Intestinal Flora

Beta diversity analysis was used to compare differences in species diversity of a pair of inter-group samples. UniFrac uses phylogenetic information to compare species diversity among samples. The evolutionary distance between species was taken into account in the calculation results. The larger the index value, the greater the difference between samples. The UniFrac results are divided into weighted UniFrac and Unweighted UniFrac: the weighted results consider the abundance of the sequence, but the unweighted results do not consider the abundance of the sequence. The samples were clustered according to the statistical results of the differences among the samples, and the distance between the samples was calculated to judge the similarity of the species composition among the samples [17].

(a) Anosim Results

**Figure 6:**
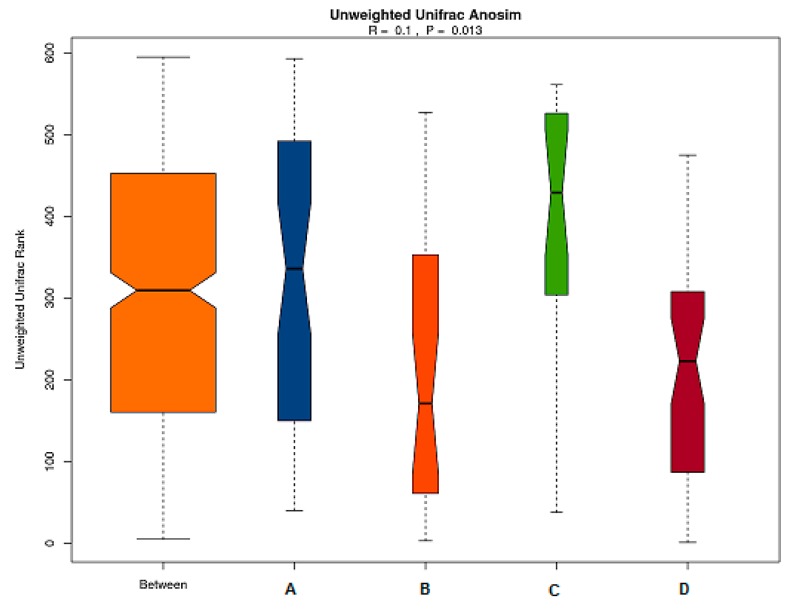
Anosim analysis of gut microbiota based on unweighted unifrac distance. The abscissa represents all samples and each grouping, and the ordinate represents the rank of the UniFrac distance. R was between (−1,1): if *R* > 0, which was a significant difference of inter-groups, whereas if *R* < 0, the difference of intra-group was greater than that of inter-groups. The reliability of the statistical analysis is expressed with *P*, and *p* < 0.05 indicates that the statistics are significant.

Anosim similarity analysis is a non-parametric test to determine whether differences of inter-groups are significantly greater than differences of intra-groups and to determine whether the grouping is meaningful. As shown in Figure 6, the R value of the unweighted results was *R* = 0.1 > 0 and *p* = 0.013 < 0.05, which indicated that the differences of inter-groups were significantly greater than that of intra-group. Thus, the grouping was reasonable, and the data were of statistical significance.

(b) PCoA Analysis Results

**Figure 7:**
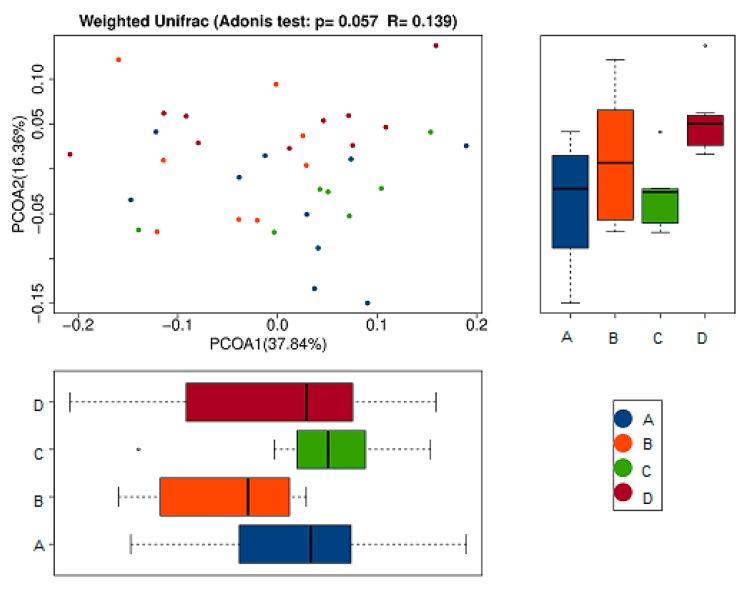
Principal coordinates analysis (PCoA) analysis of gut microbiota based on Weighted Unifrac distance. It illustrated the difference in the microbial composition among the samples. The abscissa represents the first principal coordinate, and the percentage in parentheses represents the contribution rate of the first principal coordinate to the sample difference; the ordinate represents the second principal coordinate, and the percentage in parentheses represents the contribution rate of the second principal coordinate to the sample difference.

To further compare species diversity differences among samples, principal coordinates analysis (PCoA) was used to assess the differences among samples [18,19,20]. The results of the PCoA analysis of species diversity between samples were shown in the following figure. If two samples are close distance-wise, the species compositions of the two samples are similar. As can be seen from the first principal component in Figure 7, its percentage in the model group (B) was obviously lower than that of the other groups. This indicated the intestinal flora of mice inoculated with tumor cells without any treatment was significantly different from that of the normal control group and the RPE and CP treatment groups. Moreover, the treatment groups (C and D) were close in distance to the normal control group (A), which indicated that their intestinal flora tended to be a certain similarity to the normal group (A). Additionally, in the comparison of the second principal component, the RPE treatment group (C) was also closer to the normal control group (A) than to the model group (B). It demonstrated that the intestinal flora of tumor-bearing mice tended to be more normal after RPE dietary treatment, which is a positive sign for its use in the nutritional intervention of diseases.

#### 2.2.3. Difference Analysis

**Figure 8:**
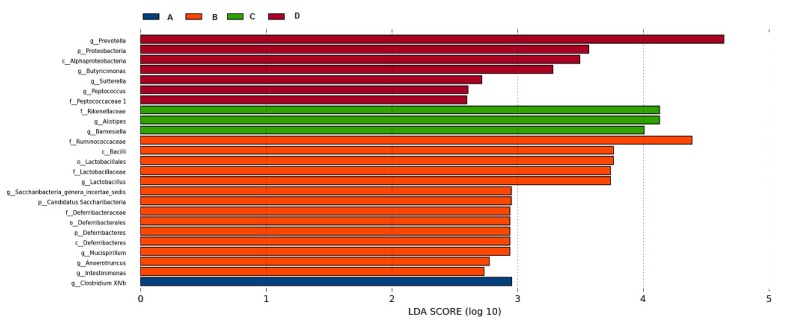
The LDA score obtained from LEfSe analysis of gut microbiota in different groups. An LDA effect value of more than 2 was used as a threshold for the LEfSe analysis.

LEfSe (LDA EffectSize) uses linear discriminant analysis (LDA) to estimate the impact of the abundance of each component, and to identify the communities or species that have significant difference effect on the division of samples. It emphasizes statistical significance and biological relevance [17]. According to species richness, the significant difference in intestinal microflora (seen Figure 8) was found in the following genera: *Clostridium XlVb* (A); *Lactobacillus, Saccharibacteria_genera_incertae_sedis, Mucispirillum, Anaerotruncus; Intestinimonas* (B); Alistipes, *Barnesiella* (C); *Prevotella, Butyricimonas, Sutterella, Peptococcus* (D).

#### 2.2.4. Species Abundance

**Figure 9:**
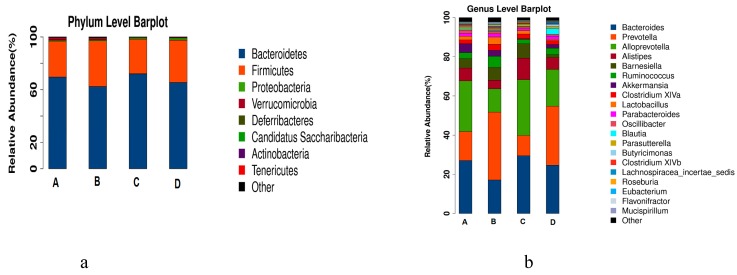
Taxonomic profiles of the fecal bacteria in the four groups at the phylum level (**a**) and genus level (**b**). The colors correspond to the names of different species, and the length of the color bar represents the proportion of relative abundance of the species.

Using the species annotation results, the corresponding histograms of species profiling were generated for each sample at the phylum, class, order, family, and genus classification levels. A histogram of the relative abundance of species can be used to identify species with a higher relative abundance in each group and individual sample at different classification levels [21]. At the phylum level, as shown in Figure 9a, different treatments mainly affected the abundance of *Bacteroides* and *Firmicutes*, which accounted for 97% of the total bacteria (shown in Table 1). Compared with that in the normal control group (A), the abundance of *Bacteroidetes* in the model group (B) decreased and that of *Firmicutes* increased; the abundance of *Bacteroidetes* in the RPE treatment groups (C) increased, and that of *Firmicutes* decreased, which was close to the normal control group (A). Notably, the changing trend of the two phyla of bacteria in the CP control group (D) was similar with the model group (B).

At the genus level, as shown in Figure 9b, according to the abundance, the top 10 groups were *Bacteroides, Prevotella, Alloprevotella, Alistipes, Barnesiella, Ruminococcus, Akkermansia, Clostridium XlVa, Lactoctococcus, and Parabacteroides*. The first six genera accounted for around 80% of the abundance in most of the groups (shown in Table 2). Compared with the normal control groups, the abundances of *Bacteroides, Alloprevotella, Alistipes, and Ruminococcus* in the model group (B) decreased, and those of *Prevotella* and *Barnesiella* increased, while the change trends of the RPE treatment group (C) were contrary to that of the model group (B), which showed that the intestinal flora of mice treated with RPE tended to be closer to that of the normal control group. In detail, compared with the normal control group (A), the abundance of *Bacteroides, Allpprevotella, Alistipes, and Ruminococcus* in the model group (B) decreased by 9.93%, 13.90%, 2.18%, and 3.46%, respectively; accordingly, compared with the model group (B), those of the RPE group (C) increased by 12.38%, 16.55%, 6.61%, and 0.91%, displaying a significant reversion to the reduction of beneficial bacteria in the model group. On the contrary, compared with the normal control group (A), the abundance of the harmful bacteria of *Prevotella* and *Barnesiella* in the model group (B) increased 19.81% and 2.56%, respectively, while those of the RPE treatment (C) decreased 24.27% and 0.79%, compared with the model group. The above results showed that RPE treatment balanced the relative abundance of beneficial bacteria and detrimental bacteria in intestinal flora, and made the total abundance of the top six genera quite closer to that of the normal control group.

## 3. Discussion

### 3.1. Tumor Inhibition

According to the data of the ratio (tumor weight/body weight) in Figure 1, the RPE treatment group (C) was significantly lower than the model group(B), and the CP control group (D) was the lowest. This illustrated that: (1) RPE treatment could significantly inhibit the growth of tumors; (2) Cyclophosphamide (CP), as a chemotherapeutic drug, had a more obvious antitumor effect.

The effect of RPE and CP on tumor inhibition could be explained in two aspects: on the one hand, the time of tumor occurrence in these two groups of mice was 2 days later than that in the model group, indicating that under the same external stimulation (inoculated the same concentration of tumor cells),with the treatment of RPE by gavage or CP by intraperitoneal injection, the occurrence of tumor could be delayed (i.e., the latency could be prolonged); on the other hand, the growth rate of tumor in the treatment groups (C and D) was slowed down, as seen from Figure 1, and the weight of the tumor in the treatment groups was evidently lower. Especially in the RPE group, as the body weight increased obviously (seen Figure 2), the ratio (of tumor weight/body weight) decreased significantly, which revealed that growth rate of the tumor was slow, without affecting the feeding and weight gain of the mice.

However, we noticed that the body weight growth of the mice in the CP control group (D) was quite inapparent (Figure 2). The main reason may be the effect of intraperitoneal injection of cyclophosphamide on their appetite inhibition or digestive capacity reduction. As a chemotherapeutic drug, cyclophosphamide has an outstanding anti-tumor effect (as shown in Figure 1), but also has obvious side effects: two mice died during the experiment, and all the mice appeared to be less energetic in the daily lives, with their food and water intake decreasing gradually. It has been pointed out that cyclophosphamide can cause digestive system dysfunction and damage immune function [22]. These results are consistent with those of intestinal flora in mice. In contrast, the mice in the RPE treatment group all survived and were fed normally all the time, observed climbing and jumping quickly and vigorously. This demonstrated that RPE had more practical significance, not only that the way of oral administration was simpler but also the life quality of the tumor bearing animals would be greatly improved.

### 3.2. The Influence on the Gut Microbiota of the Mice

The intestinal functions of the top six genera with relative abundance (shown in Table 2) were attributed through literature. It was revealed that RPE could restore the damage of intestinal flora balance in mice caused by subcutaneous inoculation of tumor cells by improving the abundance of probiotics and reducing the abundance of harmful bacteria significantly.

The main harmful bacteria was *Prevotella*, a non-spore-forming gram-negative anaerobic bacteria recently separated from *Bacteriaceae*. Clinically, as a common conditional pathogen, it can cause endogenous infections in some parts of the body, which are related to the decomposition of connective tissue. The results shown in Table 2 indicated that the abundance of intestinal flora in this genus significantly increased in mice of the model group (B), and was returned to the normal level by the treatment with RPE (C), which tended to be closer to the normal group (A). Moreover, it is worth noting that there was no significant difference between the CP control group (D) and the model group (B), which was likely because cyclophosphamide can cause endogenous infections and have an adverse effect on the structure of intestinal flora.

More beneficial bacteria were in the top six genera, as follows:

According to the literature, beneficial bacteria can maintain the balance between the body and normal flora by killing pathogenic bacteria, colonizing and antagonizing harmful bacteria, and competing with harmful bacteria for oxygen and nutrients, providing microbial barrier for intestinal tract and maintaining or restoring health of the body [23].

*Bacteroides*, a distinctive anaerobic, non-spore-forming, and bile-resistant gram-negative bacterium, is the main member of intestinal flora and plays an important role in intestinal balance. *Bacteroides* mainly use sugars as their primary energy source. They break down the complex polysaccharides in the intestine into simple sugars, which is conducive to their absorption and utilization by other bacteria [24]. They can directly inhibit the adhesion and invasion of other harmful bacteria through their own colonization [25]. When the flora becomes unbalanced, the re-introduction of *Bacteroides* can quickly restore the microbial system in the intestinal tract of the host to a balanced state [26]. It is also reported *Bacteroides* and their metabolites are closely related to tumors. They persist in the intestine because of their special structure, sphingolipid [27]. Sphingomyelin, ceramide phosphoethanolamine, sphingomyelin, and ceramide accounted for 50% of the total lipid extracts. Its different pathways have opposite physiological functions, thus, its metabolic imbalance can lead to cellular circulatory disorders. The most important is to regulate cell apoptosis through mitochondrial pathway: for example, to make the mitochondrial inner membrane produce space proteins such as cytochrome C and Apaf1, activate cysteine protease, and ultimately cause cell apoptosis [28]. In addition, ceramide, one of the major metabolites, can regulate cell proliferation, differentiation, apoptosis and inhibit tumors [29]. Xu [30] studied the effect of phcoerythrin (PE) extracted from *Gracilaria lemaneiformis* to the transcription of p53 and Caspese-3 gene in the H22-bearing mice by semi-quantitary RT-PCR (Real-time Polymerase Chain Reaction). The results showed that in vivo mRNA of p53 and Caspese-3 gene had been over-expressed, which revealed that PE can promote the expression of tumor suppressor gene, induce apoptosis of tumor cells and inhibit the growth of tumor cells. Based on the above possible mechanism, it can be inferred that the antitumor effect of phycoerythrin may be related to the effect of beneficial flora at a certain point in some anti-tumor or apoptosis pathways.

*Alloprevolella* is good at decomposing and utilizing the protein in intestinal mucus to produce folic acid and vitamin B1 [31]. The final metabolites of its fermented carbohydrates are acetic acid and succinic acid, which are conducive to maintaining intestinal homeostasis. *Alistipes*, a member of the *Bacteriodia*, is a butyric acid producing bacteria by fermentation of lysine and has been shown to be closely associated with the colitis and liver diseases in murine models and humans, respectively [32]. It is a probiotic group with similar functions as bifidobacteria, which can regulate energy metabolism in mice and help adapt to the surrounding environment [33]. Chung [34] demonstrated that the appropriate proportion of *Alistipes* may contribute to intestinal immune maturation. *Ruminocuccus* belongs to butyrate producing bacteria. Butyrate can directly provide energy for intestinal epithelium, improve intestinal digestion and absorption of nutrients, and improve intestinal immunity [35]. These probiotics can effectively inhibit the growth of pathogenic microorganisms by inhibiting the adhesion and invasion of pathogenic microorganisms to intestinal epithelial cells and competing with pathogenic microorganisms after colonization in the intestine.

## 4. Conclusions and Expectation

From the above results of tumor inhibition, dietary RPE can significantly reduce the ratio of (tumor weight/body weight), prolong the latency of tumors and inhibit the growth of tumors in H22-bearing mice. By the analysis of alpha diversity, during the selected experimental period, there was no significant difference in the composition of bacterial flora; thus, it can be concluded that the structure of gut microbiota among the four groups of mice was relatively stable. Based on the analysis of beta diversity, difference analysis, and species abundance, dietary RPE can modulate the abundance of intestinal flora of the H22-bearing mice by improving the abundance of probiotics and reducing the abundance of harmful bacteria significantly.

It is well known that dysbacteriosis is prone to chronic inflammation, while chronic inflammation is prone to tumors [36]. Intestinal flora-mediated immune response can be associated with the occurrence and development of tumors by affecting T cells, neutrophils, and related inflammatory factors [37]: metabolic products of harmful bacteria in bacterial flora can act on immune factors, stimulate secretion of inflammatory factors and tumor-related factors [38], destroy or dissolve intestinal epithelial cell membranes, change permeability [39], and intracellular toxins will enter the body. Some beneficial bacteria, if they significantly increase in number, can inhibit the migration of Th17 cells to the liver and promote Treg cells’ differentiation, and thus, inhibit the occurrence of inflammation [40]. RPE can significantly reduce the abundance of detrimental bacteria and increase the abundance of beneficial bacteria, which may be the anti-tumor effect of RPE on tumor-bearing mice through the immune regulation of intestinal flora.

Among many factors affecting the structure and abundance of intestinal flora, oral administration is relatively easy to control or change [41,42]. There is a preference or adaptability of intestinal flora to oral components [43]. For example, in the group with RPE intervention, the abundance of intestinal flora is significantly different from that of other groups, indicating that RPE may have specific functions [44]. This reflects the adaptability of intestinal flora to different substrates and also reveals the possibility of oral intervention on intestinal flora, regulation of flora structure and abundance balance and disease treatment.

Phycobiliprotein is an important light-harvesting pigment with a bright color, broad application prospects, and a variety of physiological activities. Because of its unique properties, it is widely used in foods, health products, cosmetics, biomedicine, fluorescent dyes, and other fields. In recent years, great progress has been made in the study of phycobiliproteins, showing that phycobiliproteins have anti-inflammatory, human immunity-enhancing, anti-oxidation, cell proliferation-promoting, and anti-cancer effects [45].

The intestinal tract is the largest and most important site for bacterial colonization. It participates in many physiological processes, including food degradation, nutrient absorption, and the fine-tuning of immune function [4]. Since the beginning of this century, with the in-depth development of genomics, microbiology, metabolomics, and other disciplines, an increasing number of studies have found that the imbalance of the intestinal microecological structure is closely related to the occurrence of many diseases, such as acute and chronic enteritis, coronary heart disease, diabetes, malignant tumors, and immune system diseases [46]. The intestinal flora plays an important role in nutritional metabolism, pathogen resistance, and immune system development [47]. Disruption of the intestinal flora can lead to diseases in the host. Studies have shown that a high concentration of protein can promote the growth of intestinal bacteria. On the contrary, intestinal bacteria can utilize undigested proteins and then ferment these dietary nutrients to produce metabolites such as short chain fatty acids (SCFA), which can be used as dyes for colon epithelial cells and induce intestinal cell proliferation. Thus, they are beneficial to human health [48].

In this study, different concentrations of recombinant phycobiliprotein were administered by gavage to mice inoculated with H22 hepatocellular carcinoma cells. The feces were collected aseptically on the last day of the experiment. High-throughput sequencing (macrogenome) was used to extract all microbial DNA from the samples and construct a macrogenomic library, and a genomics research strategy was used to study all microbes contained in the environmental samples to determine the genetic composition and community function.

The results showed that probiotics in intestinal flora decreased and harmful flora increased in the mice inoculated with H22 hepatoma cells. The recombinant phycobiliprotein (RPE) treatment could modulate the gut microbiota by increasing the abundance of beneficial bacteria in intestinal flora and decreasing the abundance of detrimental bacteria, which provides some basic data for the study of disease and microecology, and represent a possible approach for the treatment and improvement of some diseases based on microecology.

It is believed that with the progress of biotechnology, such as macrogenomics and metabonomics, people will have a better understanding of intestinal flora and the interaction between food or drugs and intestinal flora. It is possible to improve or treat diseases by reasonable food or drug intervention, shaping, or rebuilding rational intestinal flora.

The RPE studied in this paper can regulate the intestinal flora of tumor-bearing mice after oral administration, but the specific mechanism is still yet to be elucidated. For example, further studies are needed to clarify whether its activity is determined by the whole protein or its metabolites and how the metabolites of proteins play different roles in different microflora in vivo. With the development of metabonomics, the study of protein metabolic pathways may shed more light on their roles, which will further deepen and expand the research on phycoerythrin, its anti-tumor mechanism, and its effects on the gut microbiota.

## 5. Materials and Methods

### 5.1. Strains and the Recombinant Phycobiliprotein (RPE)

*E. coli* BL21 (DE3) and related gene source strains containing the expression plasmid pRSF-G113391-cpcS were constructed and preserved by the Key Laboratory of Experimental Marine Biology, Chinese Academy of Sciences.

Recombinant phycoerythrin (RPE) was purified by affinity chromatography after the plasmid was transformed into *E. coli* and fermented under optimized fermentation conditions [8].

### 5.2. Laboratory Animals and Treatment

All animal procedures were performed in accordance with the Guidelines for Care and Use of Laboratory Animals of Ocean University of China and experiments were approved by the Ethical Committee of Experimental Animal Care at Ocean University of China (Approval No. OUC-SMP-2019-06-01). A total of 48 specific pathogen-free mice (4–5 weeks old, male) were purchased from the Vital River Laboratory Animal Technology Co. Ltd. All mice were housed in a well-controlled environment (22–23 °C, 12 h light/dark cycles). After a 1 week adaptation period, all mice were randomly allocated into four test groups (12 in each group): normal control group (A), model group (B), RPE treatment group (C), and CP control group (D). The RPE treatment group (C) was administered RPE at 100 mg· kg bw^−1^·day^−1^. RPE was administered by gavage, and the control group (A) and model group (B) were administered an equal volume of normal saline. Mice in the (B), (C), and (D) groups were subcutaneously inoculated with 0.2 mL of an H22 cell suspension under the right forelimb axilla. The CP control group (D) was injected with cyclophosphamide (40 mg/kg) into the abdominal cavity every 2 days. Body weight was measured every 3 days after tumor challenging. After 2 weeks of treatment, all of mice of the four groups were sacrificed. Blood samples were collected from the orbital plexus and centrifuged at 1200 rpm for 20 min to separate the serum. Twenty-four hours after the last administration, the mice were weighed, and fresh feces were aseptically collected within 12 h and quickly transferred into sterile freezing tubes and stored in a freezer at −80 °C until analyzed. The mice fasted, stayed overnight, anesthetized and killed by taking blood from their eyeballs. Tumor, spleen, and liver were dissected completely and weighed accurately. Effect of tumor inhibition was calculated by tumor weight/body weight.

### 5.3. DNA Preparation and 16S rDNA High-Throughput Sequencing

The metagenomic DNA was extracted from the feces using a QIAamp Fast DNA Stool Mini Kit (Qiagen, Hamburg, Germany, No. 51604) according to the manufacturer’s instructions Specific primers with barcodes were synthesized according to the designated sequencing region. A KAPA HiFi Hotstart ReadyMix PCR Kit was used for the PCR analysis.

A pair of universal primers [forward primer (5′-3′) CCTACGGGRSGCAGCAG(F341); reverse primer (5′-3′) GGACTACVVGGGTATCTAATC(R806)] were then applied to specifically amplify the V3-V4 hypervariable regions of the 16S rDNA gene using a previously described method. The amplicons were purified and quantified before being sequenced on the HiSeq 2500 PE250 Amplifier sequencing platform (Illumina, San Diego, CA, USA) by a commercial company (Realbio Technology Co. Ltd., Shanghai, China) [49].

Recovery of 16S PCR Amplified Products and Detection of Library Size: after amplification of PCR products, PCR products were detected by 2% agarose gel electrophoresis, and AxyPrep DNA gel extraction kit was used to recover from PCR products according to the instructions. The amplified products of PCR were purified by agarose gel electrophoresis, and the library concentration was detected by NanoDrop. The size of the library was about 600 BP by 1% agarose gel electrophoresis.

Library Concentration Detection: the purified PCR product library was tested with Qubit dsDNA HS Assay Kit, and then mixed in proportion according to the requirement of each sample. Amplifier sequencing platform HiSeq 2500 PE250 was used for sequencing.

### 5.4. Bioinformatics and Sequencing Data Analysis

Effective data statistics: Paired-End sequencing through Illumina platform, Paired-End Reads grow Reads through the Overlap relationship between Reads, and quality control of the spliced Reads, get Clean Reads.

Data optimization statistics: Pandaseq software uses overlapping relationship to splice pairs of Reads from two-terminal sequencing into a sequence, and get long Reads of high-variable region. Then, the spliced Reads are processed as follows by using the program written in-house to obtain Clean Reads. (1) Removal of Reads with an average weight of less than 20; (2) Removal of Reads with a base number of more than 3 N; (3) Removal of Reads with a length of 200–500 NT.

Usearch is used for OTU (Operational Taxonomic Units) clustering under 0.97 similarity, and the clustered sequence is filtered by chimera to obtain OTU (Operational Taxonomic Units) for species classification. Finally, all Clean Reads are compared to OTU, and the Reads of OTU that can be compared are extracted. Get the final apped Reads.

A total of 524 OUTs were obtained from 50 samples of all the groups. The valid data were clustered by taxon, OTUs abundance and Alpha/Beta diversity analysis [9]. The most abundant sequence was selected from each OTU as the representative sequence. RDP method was used to compare the representative sequence with the 16S database of known species (RDP, http://rdp.cme.msu.edu). Species were classified and annotated, and each sample was annotated with each classification level (Phylum, Class, Order, Family, etc.).

### 5.5. Statistical Analysis

SPSS (Statistic Package for Social Science) statistical software was used for statistical analysis. The above data were performed by one-way ANOVA (Analysis of Variance) to compare the mean of multiple groups. The data were expressed as mean ± standard deviation [49].

Alpha diversity index analysis was aimed at single sample diversity analysis, reflecting the abundance and diversity of microbial communities. Among them, the observed_species index and Chao1 index were used to calculate the abundance of bacteria; Shannon index, Simpson index and PD_whole_tree index were used to calculate the diversity of bacteria.

Beta diversity reflects the differences among different grouping samples. UniFrac was analyzed by systematic information, which was divided into weighted UniFrac (Weighted UniFrac) results and non-weighted UniFrac (Unweighted UniFrac) results. The distribution of each sample in each taxon can be visualized by NMDS (Non-metric Multidimentional scaling) analysis. Anosim analysis, PCoA analysis, LefSe difference analysis, rank between groups and other methods were used to analyze the difference significance.

## Figures and Tables

**Table 1:** The relative abundance of the main gut microbiota at the phylum level in four groups.

	Abundance (%)			
	A	B	C	D
*Bacteroidetes*	69.55251	62.32627	71.99758	65.44234
*Firmicutes*	27.29629	34.71815	26.42178	32.09082
Total	96.8488	97.04442	98.41936	97.53316

Note: The abundance of *Bacteroides* and *Firmicutes* accounted for 97% of the total bacteria. The abundance of the two phyla of the model group (B) and the treatment groups (C and D) showed obvious changes.

**Table 2:** The relative abundance of the main gut microbiota at the genus level in four groups.

	Abundance (%)			
	A	B	C	D
*Bacteroides*	27.04936	17.1156	29.49336	24.68184
*Prevotella*	14.79315	34.60532	10.33851	30.07564
*Alloprevotella*	25.83259	11.93675	28.48872	18.76582
*Alistipes*	6.529005	4.347667	10.96018	5.97906
*Barnesiella*	4.812605	7.369688	6.575657	1.543275
*Ruminococcus*	5.674671	2.21009	3.120995	3.329431
Total	84.69138	77.58512	88.97742	84.37507

**Note:** The top six genera accounted for around 80% of the abundance in most of the groups. The abundance of the six genera of the model group (B) and the treatment groups (C and D) showed obvious changes.
